# Supplementary material for: State-Level Prevalence and Associates of Opioid Dependence in the USA
Source: Int J Environ Res Public Health. 2022 Mar 23;19(7):3825. doi: 10.3390/ijerph19073825 (PMC8997413; doi:10.3390/ijerph19073825)
Supplement: Supplementary file 1 [file ijerph-19-03825-s001.zip › ijerph-1535104-supplementary.pdf]

Supplementary Material

State-Level Prevalence and Associates of Opioid Dependence in the USA

Janni Leung*, Gary C. K. Chan, Samuel X. Tan, Caitlin McClure-Thomas, Louisa Degenhardt, Wayne Hall (2022).

* Correspondence: author: National Centre for Youth Substance Use Research, The University of Queensland

Supplement S1: State-level prevalence of opioids dependence in the past year in National Survey on Drug Use and Health (NSDUH) 2005-2014

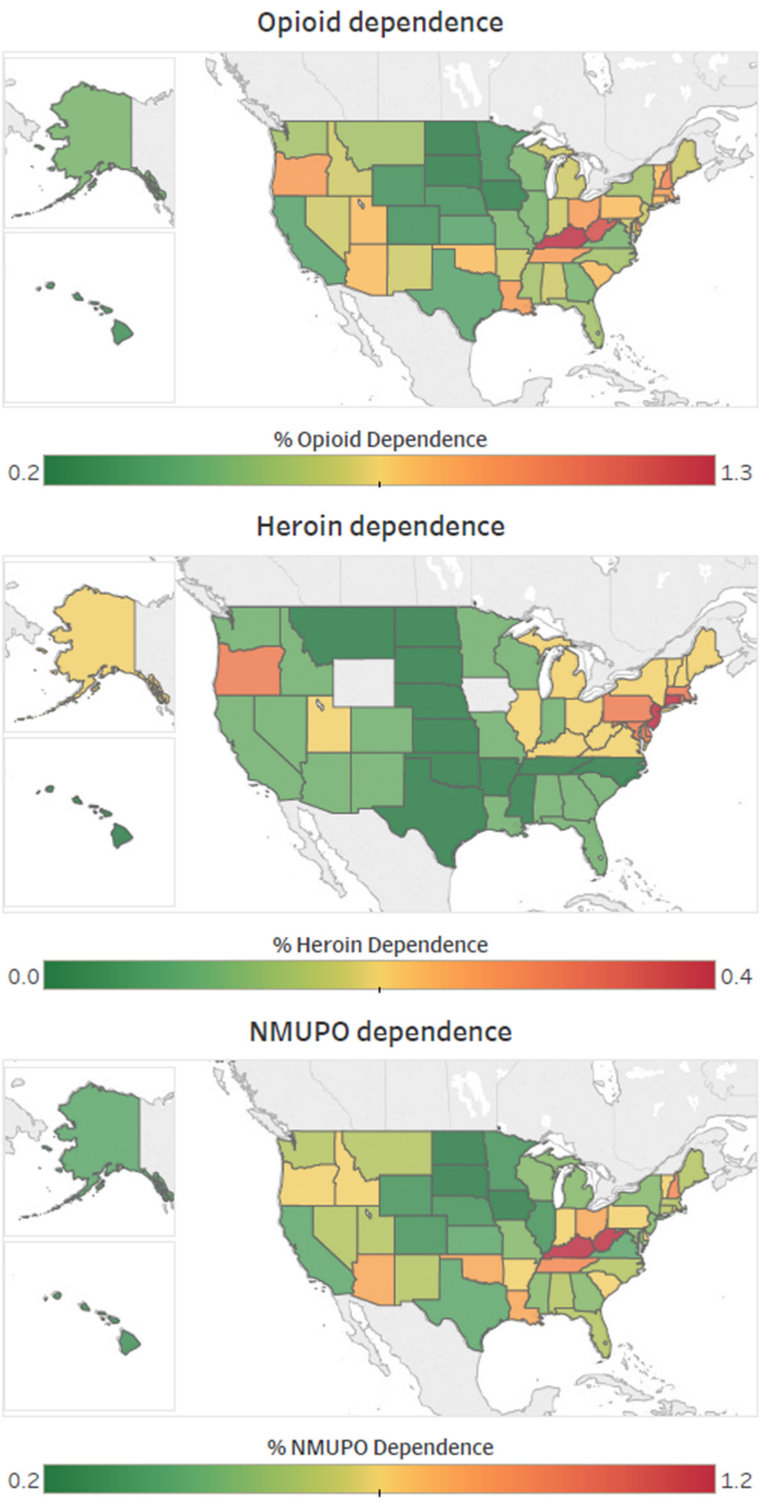

Figure S1. State-level prevalence of opioids dependence, heroin dependence, and NMPO dependence.

Footnote for data tables

*Low precision; no estimate reported.

NOTE: Dependence is based on the definition found in the 4th edition of the Diagnostic and Statistical Manual of Mental Disorders (DSM-IV).

Source: SAMHSA, Center for Behavioral Health Statistics and Quality, National Survey on Drug Use and Health, 2005-2014. Weights were used to account for selection probability, response rate, and demographic representation.

Table S1. State-level prevalence of opioids (heroin or non-medicinal use of prescription opioids [NMPO]) dependence in past year among persons aged 18 or older, by age group and sex: percentages, and 95% confidence intervals, averages based on 2005-2014.

State	Past year opioids (heroin or NMPO) dependence % (95%CI)					
	Age groups			Aged 18+		
	Aged 18-25	Aged 26-49	Aged 50+	Both sex	Male	Female
	% (CI)	% (CI)	% (CI)	% (CI)	% (CI)	% (CI)
Alabama	1.5 (1.0-2.1)	1.0 (0.6-1.5)	0.2 (0.0-1.2)	0.7 (0.5-1.0)	1.1 (0.7-1.6)	0.4 (0.2-0.6)
Alaska	1.4 (1.0-2.0)	0.4 (0.2-0.8)	0.1 (0.0-1.0)	0.5 (0.3-0.7)	0.6 (0.4-1.1)	0.4 (0.2-0.6)
Arizona	1.4 (0.9-2.1)	0.9 (0.6-1.5)	0.5 (0.2-1.4)	0.8 (0.6-1.2)	1.1 (0.7-1.8)	0.5 (0.3-0.9)
Arkansas	1.5 (0.9-2.3)	0.9 (0.5-1.4)	0.2 (0.0-1.5)	0.7 (0.4-1.0)	0.9 (0.5-1.6)	0.4 (0.2-0.7)
California	1.2 (0.9-1.4)	0.5 (0.4-0.7)	0.1 (0.0-0.2)	0.4 (0.4-0.6)	0.6 (0.4-0.7)	0.3 (0.3-0.5)
Colorado	1.2 (0.8-1.9)	0.3 (0.1-0.8)	* (*-*)	0.3 (0.2-0.5)	0.5 (0.3-0.9)	0.2 (0.1-0.4)
Connecticut	2.4 (1.9-3.1)	0.7 (0.4-1.4)	0.3 (0.1-1.1)	0.8 (0.6-1.1)	1.1 (0.7-1.7)	0.5 (0.3-0.8)
Delaware	2.9 (2.2-3.9)	1.1 (0.6-1.8)	0.1 (0.0-0.3)	0.9 (0.6-1.2)	0.9 (0.6-1.4)	0.9 (0.6-1.3)
District of Columbia	0.5 (0.3-0.9)	0.2 (0.1-0.7)	0.8 (0.3-2.1)	0.5 (0.3-0.9)	0.8 (0.4-1.7)	0.3 (0.1-0.6)
Florida	1.5 (1.2-1.9)	0.7 (0.5-0.9)	0.3 (0.2-0.5)	0.6 (0.5-0.7)	0.9 (0.7-1.1)	0.4 (0.3-0.5)
Georgia	1.1 (0.7-1.6)	0.5 (0.2-1.2)	0.2 (0.1-0.7)	0.5 (0.3-0.8)	0.5 (0.3-0.8)	0.5 (0.2-1.0)
Hawaii	0.4 (0.2-0.9)	0.5 (0.2-1.0)	0.1 (0.0-0.5)	0.3 (0.2-0.5)	0.3 (0.1-0.7)	0.3 (0.1-0.6)
Idaho	1.7 (1.3-2.2)	0.9 (0.6-1.5)	0.2 (0.1-1.0)	0.7 (0.5-1.1)	0.4 (0.3-0.7)	1.0 (0.7-1.6)
Illinois	0.8 (0.6-1.0)	0.8 (0.6-1.0)	0.2 (0.1-0.4)	0.5 (0.4-0.7)	0.7 (0.5-0.9)	0.4 (0.3-0.5)
Indiana	1.8 (1.3-2.5)	0.6 (0.3-1.1)	0.5 (0.2-1.4)	0.7 (0.5-0.9)	0.8 (0.5-1.2)	0.6 (0.4-0.9)
Iowa	0.5 (0.3-0.9)	0.2 (0.1-0.5)	0.2 (0.0-0.8)	0.2 (0.1-0.5)	0.3 (0.1-0.8)	0.2 (0.1-0.4)
Kansas	0.7 (0.4-1.2)	0.5 (0.2-0.9)	0.2 (0.0-0.7)	0.4 (0.2-0.6)	0.4 (0.2-0.7)	0.4 (0.2-0.8)
Kentucky	2.8 (2.2-3.6)	1.9 (1.4-2.5)	0.2 (0.0-0.8)	1.3 (1.0-1.6)	1.5 (1.1-2.1)	1.0 (0.7-1.5)
Louisiana	1.4 (1.1-1.9)	1.3 (0.9-1.8)	0.2 (0.0-1.6)	0.9 (0.6-1.2)	1.1 (0.7-1.7)	0.7 (0.4-1.0)
Maine	2.7 (2.1-3.6)	0.9 (0.5-1.6)	* (*-*)	0.7 (0.5-1.0)	0.8 (0.6-1.1)	0.6 (0.4-1.0)
Maryland	1.7 (1.2-2.5)	0.8 (0.5-1.2)	0.2 (0.1-0.7)	0.7 (0.5-0.9)	1.0 (0.7-1.4)	0.4 (0.2-0.6)
Massachusetts	2.0 (1.5-2.8)	1.0 (0.7-1.6)	0.4 (0.1-1.3)	0.9 (0.6-1.3)	1.4 (0.9-2.1)	0.5 (0.3-0.7)
Michigan	1.4 (1.1-1.6)	0.8 (0.6-1.1)	0.3 (0.2-0.5)	0.7 (0.6-0.8)	0.8 (0.6-1.0)	0.6 (0.5-0.7)

State	Past year opioids (heroin or NMPO) dependence % (95%CI)					
	Age groups			Aged 18+		
	Aged 18-25	Aged 26-49	Aged 50+	Both sex	Male	Female
	% (CI)	% (CI)	% (CI)	% (CI)	% (CI)	% (CI)
Minnesota	0.7 (0.4-1.0)	0.4 (0.2-0.8)	0.1 (0.0-0.7)	0.3 (0.2-0.5)	0.5 (0.2-0.9)	0.2 (0.1-0.4)
Mississippi	1.5 (1.1-2.0)	0.6 (0.3-1.1)	0.2 (0.1-0.8)	0.6 (0.4-0.8)	0.7 (0.5-1.0)	0.5 (0.3-0.9)
Missouri	1.6 (1.1-2.4)	0.6 (0.3-1.0)	0.1 (0.0-0.6)	0.5 (0.4-0.7)	0.6 (0.4-0.9)	0.5 (0.3-0.7)
Montana	1.5 (1.0-2.2)	0.9 (0.5-1.5)	* (*-*)	0.6 (0.4-0.8)	0.5 (0.3-0.8)	0.6 (0.4-1.0)
Nebraska	0.9 (0.6-1.5)	0.2 (0.1-0.5)	0.2 (0.0-1.2)	0.3 (0.2-0.5)	0.3 (0.1-0.5)	0.4 (0.1-0.9)
Nevada	2.0 (1.3-3.1)	0.9 (0.5-1.4)	0.2 (0.0-0.7)	0.7 (0.5-1.0)	0.7 (0.5-1.1)	0.7 (0.5-1.1)
New Hampshire	2.0 (1.5-2.6)	1.2 (0.8-1.8)	0.4 (0.1-1.2)	1.0 (0.7-1.4)	1.2 (0.8-1.9)	0.7 (0.5-1.1)
New Jersey	1.8 (1.3-2.5)	0.9 (0.6-1.4)	0.2 (0.1-0.6)	0.7 (0.5-1.0)	0.8 (0.6-1.2)	0.6 (0.4-0.9)
New Mexico	1.4 (1.0-2.1)	0.8 (0.5-1.3)	0.4 (0.1-1.7)	0.7 (0.5-1.1)	1.0 (0.5-1.8)	0.4 (0.3-0.6)
New York	1.2 (1.0-1.4)	0.7 (0.6-0.9)	0.3 (0.2-0.6)	0.6 (0.5-0.8)	0.9 (0.7-1.1)	0.4 (0.3-0.6)
North Carolina	1.5 (1.2-2.1)	0.5 (0.3-0.9)	0.3 (0.1-1.4)	0.6 (0.3-0.9)	0.9 (0.5-1.7)	0.3 (0.2-0.5)
North Dakota	0.7 (0.4-1.1)	0.2 (0.1-0.4)	* (*-*)	0.2 (0.1-0.3)	0.2 (0.1-0.3)	0.2 (0.2-0.4)
Ohio	1.8 (1.5-2.1)	1.2 (1.0-1.4)	0.3 (0.2-0.5)	0.9 (0.7-1.0)	0.9 (0.8-1.1)	0.8 (0.6-1.0)
Oklahoma	1.3 (0.9-1.9)	1.1 (0.8-1.6)	0.4 (0.1-1.8)	0.8 (0.6-1.2)	1.1 (0.7-1.7)	0.6 (0.3-1.2)
Oregon	1.7 (1.2-2.5)	1.3 (0.8-2.0)	0.2 (0.1-1.0)	0.9 (0.6-1.2)	1.2 (0.8-1.9)	0.6 (0.4-0.9)
Pennsylvania	2.2 (1.8-2.5)	1.0 (0.8-1.3)	0.3 (0.1-0.5)	0.8 (0.7-1.0)	1.0 (0.8-1.2)	0.7 (0.5-0.9)
Rhode Island	1.5 (1.0-2.1)	1.5 (1.0-2.3)	0.1 (0.0-0.8)	0.9 (0.7-1.3)	1.2 (0.7-1.8)	0.7 (0.5-1.0)
South Carolina	1.3 (0.8-1.9)	0.9 (0.6-1.5)	0.4 (0.1-1.3)	0.8 (0.5-1.1)	0.9 (0.5-1.5)	0.7 (0.4-1.2)
South Dakota	0.6 (0.3-1.2)	0.1 (0.0-0.6)	0.1 (0.0-0.8)	0.2 (0.1-0.4)	0.1 (0.0-0.4)	0.2 (0.1-0.6)
Tennessee	2.4 (1.8-3.2)	1.0 (0.6-1.5)	0.3 (0.1-0.8)	0.9 (0.6-1.2)	1.1 (0.8-1.5)	0.7 (0.4-1.1)
Texas	0.8 (0.7-1.0)	0.5 (0.3-0.6)	0.1 (0.1-0.3)	0.4 (0.3-0.5)	0.4 (0.3-0.5)	0.4 (0.3-0.5)
Utah	1.7 (1.2-2.5)	0.8 (0.5-1.4)	0.1 (0.0-0.4)	0.8 (0.5-1.0)	0.8 (0.5-1.2)	0.8 (0.5-1.2)
Vermont	2.4 (1.8-3.1)	0.9 (0.6-1.4)	0.1 (0.0-0.6)	0.8 (0.6-1.0)	0.9 (0.6-1.3)	0.7 (0.4-1.1)
Virginia	1.2 (0.8-1.7)	0.5 (0.3-1.0)	0.1 (0.0-0.8)	0.5 (0.3-0.7)	0.4 (0.2-0.8)	0.5 (0.2-0.9)
Washington	1.2 (0.9-1.7)	0.9 (0.6-1.5)	0.1 (0.0-0.4)	0.6 (0.4-0.9)	0.7 (0.4-1.2)	0.5 (0.4-0.8)
West Virginia	3.6 (2.9-4.4)	1.4 (0.9-2.1)	0.4 (0.1-1.0)	1.2 (0.9-1.5)	1.4 (1.0-1.9)	1.0 (0.7-1.5)
Wisconsin	1.3 (0.8-1.9)	0.5 (0.3-0.8)	0.3 (0.1-1.2)	0.5 (0.3-0.7)	0.4 (0.3-0.7)	0.5 (0.3-1.1)
Wyoming	0.9 (0.6-1.3)	0.4 (0.2-0.8)	0.1 (0.0-0.9)	0.3 (0.2-0.5)	0.4 (0.2-0.7)	0.3 (0.1-0.7)

Table S2. State-level prevalence of past year heroin dependence among persons aged 18 or older, by age group and sex: averages based on 2005-2014.

State	Past year heroin dependence % (95%CI)					
	Age groups			Aged 18+		
	Aged 18-25	Aged 26-49	Aged 50+	Both sex	Male	Female
	% (CI)	% (CI)	% (CI)	% (CI)	% (CI)	% (CI)
Alabama	0.2 (0.1-0.5)	0.2 (0.0-0.5)	* (*-*)	0.1 (0.0-0.2)	0.2 (0.1-0.5)	0.0 (0.0-0.1)
Alaska	0.8 (0.5-1.3)	0.1 (0.0-0.5)	* (*-*)	0.2 (0.1-0.3)	0.3 (0.2-0.6)	0.1 (0.0-0.2)
Arizona	0.3 (0.2-0.6)	0.1 (0.0-0.3)	* (*-*)	0.1 (0.1-0.2)	0.1 (0.0-0.2)	0.1 (0.0-0.3)
Arkansas	0.1 (0.0-0.3)	* (*-*)	* (*-*)	0.0 (0.0-0.0)	0.0 (0.0-0.1)	0.0 (0.0-0.1)
California	0.2 (0.1-0.4)	0.1 (0.1-0.3)	0.0 (0.0-0.2)	0.1 (0.1-0.2)	0.2 (0.1-0.3)	0.1 (0.0-0.1)
Colorado	0.4 (0.2-0.9)	* (*-*)	* (*-*)	0.1 (0.0-0.1)	0.1 (0.0-0.2)	0.0 (0.0-0.1)
Connecticut	1.2 (0.7-1.9)	0.3 (0.1-0.8)	0.2 (0.0-0.9)	0.4 (0.2-0.6)	0.6 (0.3-1.0)	0.2 (0.1-0.4)
Delaware	0.9 (0.5-1.5)	0.5 (0.2-1.0)	0.0 (0.0-0.1)	0.3 (0.2-0.6)	0.4 (0.2-0.8)	0.3 (0.2-0.6)
District of Columbia	0.0 (0.0-0.2)	0.2 (0.1-0.7)	0.7 (0.2-2.0)	0.4 (0.2-0.8)	0.7 (0.3-1.6)	0.1 (0.0-0.3)
Florida	0.2 (0.1-0.4)	0.1 (0.0-0.2)	0.1 (0.0-0.2)	0.1 (0.1-0.2)	0.2 (0.1-0.3)	0.0 (0.0-0.1)
Georgia	0.3 (0.1-0.6)	0.1 (0.0-0.4)	0.1 (0.0-0.6)	0.1 (0.1-0.3)	0.2 (0.1-0.5)	0.0 (0.0-0.1)
Hawaii	0.1 (0.0-0.4)	* (*-*)	* (*-*)	0.0 (0.0-0.1)	0.0 (0.0-0.1)	* (*-*)
Idaho	0.0 (0.0-0.3)	0.2 (0.1-0.5)	* (*-*)	0.1 (0.0-0.2)	0.1 (0.0-0.3)	0.1 (0.0-0.3)
Illinois	0.2 (0.2-0.4)	0.3 (0.2-0.5)	0.1 (0.0-0.3)	0.2 (0.2-0.3)	0.3 (0.2-0.5)	0.1 (0.1-0.2)
Indiana	0.4 (0.2-0.8)	0.1 (0.0-0.5)	* (*-*)	0.1 (0.1-0.2)	0.2 (0.1-0.4)	0.1 (0.0-0.2)
Iowa	* (*-*)	* (*-*)	* (*-*)	* (*-*)	* (*-*)	* (*-*)
Kansas	0.1 (0.0-0.4)	* (*-*)	* (*-*)	0.0 (0.0-0.1)	0.0 (0.0-0.1)	* (*-*)
Kentucky	0.5 (0.2-1.0)	0.2 (0.1-0.5)	* (*-*)	0.2 (0.1-0.3)	0.1 (0.1-0.3)	0.2 (0.1-0.4)
Louisiana	0.1 (0.0-0.3)	0.3 (0.1-0.7)	* (*-*)	0.1 (0.0-0.3)	0.1 (0.0-0.5)	0.1 (0.0-0.4)
Maine	1.0 (0.6-1.6)	0.1 (0.0-0.4)	* (*-*)	0.2 (0.1-0.3)	0.2 (0.1-0.4)	0.1 (0.1-0.2)
Maryland	0.6 (0.3-1.0)	0.3 (0.2-0.7)	0.0 (0.0-0.3)	0.3 (0.2-0.4)	0.4 (0.2-0.7)	0.1 (0.1-0.3)
Massachusetts	0.4 (0.2-0.8)	0.4 (0.2-0.7)	0.3 (0.1-1.3)	0.3 (0.2-0.6)	0.5 (0.2-1.1)	0.2 (0.1-0.5)
Michigan	0.3 (0.3-0.5)	0.2 (0.1-0.4)	0.1 (0.0-0.3)	0.2 (0.1-0.3)	0.2 (0.1-0.4)	0.2 (0.1-0.3)
Minnesota	0.1 (0.0-0.3)	* (*-*)	0.1 (0.0-0.7)	0.1 (0.0-0.2)	0.1 (0.0-0.5)	* (*-*)

State	Past year heroin dependence % (95%CI)					
	Age groups			Aged 18+		
	Aged 18-25	Aged 26-49	Aged 50+	Both sex	Male	Female
	% (CI)	% (CI)	% (CI)	% (CI)	% (CI)	% (CI)
Mississippi	0.2 (0.1-0.6)	0.0 (0.0-0.3)	* (*-*)	0.0 (0.0-0.1)	0.1 (0.0-0.2)	0.0 (0.0-0.2)
Missouri	0.4 (0.2-0.7)	0.1 (0.0-0.4)	* (*-*)	0.1 (0.0-0.2)	0.1 (0.0-0.3)	0.1 (0.0-0.3)
Montana	0.1 (0.0-0.2)	* (*-*)	* (*-*)	0.0 (0.0-0.0)	0.0 (0.0-0.1)	0.0 (0.0-0.0)
Nebraska	0.2 (0.0-0.9)	* (*-*)	* (*-*)	0.0 (0.0-0.1)	0.0 (0.0-0.1)	0.0 (0.0-0.3)
Nevada	0.5 (0.2-1.7)	0.1 (0.0-0.7)	* (*-*)	0.1 (0.1-0.4)	0.1 (0.0-0.5)	0.2 (0.0-0.6)
New Hampshire	0.6 (0.4-1.1)	0.4 (0.2-0.8)	* (*-*)	0.2 (0.1-0.4)	0.3 (0.1-0.5)	0.2 (0.1-0.5)
New Jersey	1.0 (0.7-1.3)	0.5 (0.3-0.9)	0.1 (0.0-0.5)	0.4 (0.3-0.6)	0.4 (0.3-0.7)	0.3 (0.2-0.6)
New Mexico	0.5 (0.3-0.9)	0.2 (0.1-0.5)	* (*-*)	0.1 (0.1-0.2)	0.1 (0.1-0.3)	0.2 (0.1-0.4)
New York	0.3 (0.2-0.4)	0.2 (0.1-0.4)	0.2 (0.1-0.4)	0.2 (0.1-0.3)	0.3 (0.2-0.5)	0.1 (0.1-0.2)
North Carolina	0.1 (0.1-0.3)	0.1 (0.0-0.5)	* (*-*)	0.0 (0.0-0.2)	0.1 (0.0-0.3)	0.0 (0.0-0.0)
North Dakota	0.0 (0.0-0.2)	* (*-*)	* (*-*)	0.0 (0.0-0.0)	0.0 (0.0-0.1)	* (*-*)
Ohio	0.4 (0.3-0.6)	0.3 (0.2-0.4)	* (*-*)	0.2 (0.1-0.2)	0.2 (0.1-0.3)	0.2 (0.1-0.2)
Oklahoma	0.0 (0.0-0.2)	0.1 (0.0-0.4)	* (*-*)	0.0 (0.0-0.2)	0.0 (0.0-0.4)	0.0 (0.0-0.1)
Oregon	0.4 (0.2-1.1)	0.5 (0.2-1.1)	* (*-*)	0.3 (0.1-0.5)	0.4 (0.2-0.9)	0.2 (0.1-0.3)
Pennsylvania	0.9 (0.7-1.1)	0.4 (0.2-0.6)	0.1 (0.0-0.2)	0.3 (0.2-0.4)	0.4 (0.3-0.6)	0.2 (0.2-0.3)
Rhode Island	0.3 (0.1-0.7)	0.5 (0.2-1.2)	0.0 (0.0-0.1)	0.3 (0.1-0.5)	0.4 (0.2-1.0)	0.1 (0.0-0.2)
South Carolina	0.2 (0.1-0.6)	0.2 (0.1-0.6)	* (*-*)	0.1 (0.0-0.3)	0.2 (0.1-0.5)	0.0 (0.0-0.1)
South Dakota	0.1 (0.0-0.5)	* (*-*)	* (*-*)	0.0 (0.0-0.1)	* (*-*)	0.0 (0.0-0.2)
Tennessee	0.1 (0.0-0.2)	0.0 (0.0-0.3)	* (*-*)	0.0 (0.0-0.1)	0.1 (0.0-0.1)	* (*-*)
Texas	0.1 (0.1-0.2)	0.0 (0.0-0.1)	* (*-*)	0.0 (0.0-0.1)	0.0 (0.0-0.1)	0.0 (0.0-0.0)
Utah	0.8 (0.4-1.4)	0.1 (0.0-0.3)	* (*-*)	0.2 (0.1-0.3)	0.2 (0.1-0.4)	0.2 (0.1-0.4)
Vermont	0.4 (0.2-0.8)	0.2 (0.1-0.5)	* (*-*)	0.2 (0.1-0.3)	0.2 (0.1-0.4)	0.1 (0.1-0.3)
Virginia	0.3 (0.2-0.7)	0.3 (0.1-0.6)	* (*-*)	0.2 (0.1-0.3)	0.2 (0.1-0.4)	0.1 (0.0-0.5)
Washington	0.2 (0.1-0.5)	0.2 (0.1-0.5)	0.1 (0.0-0.4)	0.1 (0.1-0.3)	0.3 (0.1-0.6)	0.0 (0.0-0.1)
West Virginia	0.8 (0.5-1.2)	0.1 (0.0-0.5)	0.0 (0.0-0.3)	0.2 (0.1-0.3)	0.1 (0.1-0.3)	0.2 (0.1-0.4)
Wisconsin	0.3 (0.2-0.8)	0.0 (0.0-0.2)	* (*-*)	0.1 (0.0-0.1)	0.1 (0.0-0.2)	0.0 (0.0-0.1)
Wyoming	0.0 (0.0-0.2)	* (*-*)	* (*-*)	* (*-*)	0.0 (0.0-0.1)	* (*-*)

Table S3. Prevalence of non-medical prescription opioid (NMPO) dependence in past year among persons aged 18 or older, by age group and sex: averages based on 2005-2014.

State	Past year NMPO dependence % (95%CI)					
	Age groups			Aged 18+		
	Aged 18-25	Aged 26-49	Aged 50+	Both sex	Male	Female
	% (CI)	% (CI)	% (CI)	% (CI)	% (CI)	% (CI)
Alabama	1.4 (0.9-2.0)	0.9 (0.5-1.4)	0.2 (0.0-1.2)	0.6 (0.4-0.9)	0.9 (0.6-1.5)	0.4 (0.2-0.6)
Alaska	1.1 (0.7-1.7)	0.4 (0.2-0.7)	0.1 (0.0-1.0)	0.4 (0.3-0.6)	0.5 (0.3-0.9)	0.3 (0.2-0.5)
Arizona	1.2 (0.7-1.9)	0.9 (0.5-1.4)	0.5 (0.2-1.4)	0.8 (0.5-1.1)	1.1 (0.7-1.7)	0.5 (0.3-0.8)
Arkansas	1.4 (0.9-2.2)	0.9 (0.5-1.4)	0.2 (0.0-1.5)	0.7 (0.4-1.0)	0.9 (0.5-1.6)	0.4 (0.2-0.7)
California	1.0 (0.8-1.3)	0.4 (0.3-0.6)	0.0 (0.0-0.1)	0.4 (0.3-0.5)	0.5 (0.3-0.6)	0.3 (0.2-0.4)
Colorado	1.0 (0.6-1.5)	0.3 (0.1-0.8)	* (*-*)	0.3 (0.2-0.5)	0.4 (0.2-0.8)	0.2 (0.1-0.4)
Connecticut	1.6 (1.1-2.4)	0.5 (0.3-1.1)	0.3 (0.1-1.1)	0.6 (0.4-0.9)	0.8 (0.4-1.5)	0.4 (0.2-0.6)
Delaware	2.5 (1.8-3.4)	0.7 (0.4-1.4)	0.1 (0.0-0.4)	0.7 (0.5-1.0)	0.6 (0.4-0.9)	0.8 (0.5-1.2)
District of Columbia	0.5 (0.3-0.9)	* (*-*)	0.3 (0.1-1.0)	0.2 (0.1-0.4)	0.2 (0.1-0.5)	0.2 (0.0-0.5)
Florida	1.5 (1.2-1.8)	0.7 (0.5-0.9)	0.2 (0.1-0.5)	0.6 (0.5-0.7)	0.8 (0.6-1.1)	0.3 (0.3-0.4)
Georgia	0.9 (0.6-1.4)	0.5 (0.2-1.1)	0.2 (0.1-0.7)	0.5 (0.3-0.7)	0.5 (0.3-0.8)	0.5 (0.2-1.0)
Hawaii	0.4 (0.2-0.8)	0.5 (0.2-1.0)	0.1 (0.0-0.5)	0.3 (0.2-0.5)	0.3 (0.1-0.7)	0.3 (0.1-0.6)
Idaho	1.7 (1.3-2.2)	0.9 (0.5-1.4)	0.2 (0.1-1.0)	0.7 (0.5-1.0)	0.4 (0.3-0.7)	1.0 (0.6-1.6)
Illinois	0.7 (0.5-0.9)	0.4 (0.3-0.6)	0.1 (0.0-0.3)	0.3 (0.3-0.4)	0.4 (0.3-0.6)	0.3 (0.2-0.4)
Indiana	1.5 (1.1-2.1)	0.5 (0.3-1.0)	0.5 (0.2-1.4)	0.7 (0.5-0.9)	0.7 (0.5-1.1)	0.6 (0.4-0.9)
Iowa	0.5 (0.3-0.9)	0.2 (0.1-0.5)	0.2 (0.0-0.8)	0.2 (0.1-0.5)	0.3 (0.1-0.8)	0.2 (0.1-0.4)
Kansas	0.7 (0.4-1.2)	0.5 (0.2-0.9)	0.2 (0.0-0.7)	0.4 (0.2-0.6)	0.3 (0.2-0.7)	0.4 (0.2-0.8)
Kentucky	2.5 (1.9-3.2)	1.8 (1.3-2.5)	0.2 (0.0-0.8)	1.2 (0.9-1.6)	1.5 (1.1-2.0)	0.9 (0.6-1.4)
Louisiana	1.4 (1.1-1.9)	1.2 (0.9-1.7)	0.2 (0.0-1.6)	0.8 (0.6-1.1)	1.0 (0.7-1.6)	0.7 (0.4-1.0)
Maine	2.4 (1.9-3.2)	0.8 (0.5-1.5)	* (*-*)	0.6 (0.5-0.9)	0.7 (0.5-1.0)	0.6 (0.3-0.9)
Maryland	1.5 (1.0-2.1)	0.5 (0.3-0.9)	0.2 (0.0-0.7)	0.5 (0.4-0.7)	0.8 (0.5-1.1)	0.3 (0.2-0.5)
Massachusetts	1.8 (1.3-2.4)	0.7 (0.4-1.2)	0.2 (0.0-0.7)	0.6 (0.5-0.9)	1.0 (0.7-1.5)	0.3 (0.2-0.5)
Michigan	1.1 (0.9-1.4)	0.7 (0.5-0.9)	0.2 (0.1-0.4)	0.5 (0.4-0.6)	0.6 (0.5-0.8)	0.5 (0.3-0.6)
Minnesota	0.6 (0.3-0.9)	0.4 (0.2-0.8)	0.1 (0.0-0.4)	0.3 (0.2-0.4)	0.3 (0.2-0.7)	0.2 (0.1-0.4)

State	Past year NMPO dependence % (95%CI)					
	Age groups			Aged 18+		
	Aged 18-25	Aged 26-49	Aged 50+	Both sex	Male	Female
	% (CI)	% (CI)	% (CI)	% (CI)	% (CI)	% (CI)
Mississippi	1.3 (1.0-1.8)	0.6 (0.3-1.1)	0.2 (0.1-0.8)	0.5 (0.4-0.8)	0.6 (0.4-0.9)	0.5 (0.3-0.9)
Missouri	1.3 (0.8-2.1)	0.6 (0.3-1.0)	0.1 (0.0-0.6)	0.5 (0.3-0.7)	0.5 (0.3-0.9)	0.4 (0.2-0.7)
Montana	1.5 (1.0-2.2)	0.9 (0.5-1.5)	* (*-*)	0.6 (0.4-0.8)	0.5 (0.3-0.8)	0.6 (0.3-1.0)
Nebraska	0.9 (0.5-1.4)	0.2 (0.1-0.5)	0.2 (0.0-1.2)	0.3 (0.2-0.5)	0.2 (0.1-0.5)	0.4 (0.1-0.9)
Nevada	1.8 (1.2-2.8)	0.7 (0.4-1.2)	0.2 (0.0-0.7)	0.6 (0.5-0.9)	0.7 (0.5-1.1)	0.6 (0.4-0.9)
New Hampshire	1.8 (1.3-2.4)	1.2 (0.8-1.8)	0.4 (0.1-1.2)	0.9 (0.6-1.3)	1.1 (0.7-1.8)	0.7 (0.4-1.1)
New Jersey	1.2 (0.7-2.2)	0.5 (0.3-0.9)	0.2 (0.1-0.6)	0.5 (0.3-0.7)	0.6 (0.4-1.0)	0.3 (0.2-0.5)
New Mexico	1.1 (0.7-1.8)	0.6 (0.4-1.0)	0.4 (0.1-1.7)	0.6 (0.3-1.0)	0.9 (0.4-1.7)	0.3 (0.2-0.4)
New York	1.0 (0.8-1.2)	0.6 (0.5-0.8)	0.2 (0.1-0.4)	0.5 (0.4-0.6)	0.7 (0.5-0.8)	0.3 (0.2-0.4)
North Carolina	1.5 (1.1-2.0)	0.5 (0.3-0.9)	0.3 (0.1-1.4)	0.6 (0.3-0.9)	0.9 (0.4-1.7)	0.3 (0.2-0.4)
North Dakota	0.7 (0.4-1.1)	0.2 (0.1-0.4)	* (*-*)	0.2 (0.1-0.3)	0.1 (0.1-0.4)	0.2 (0.2-0.4)
Ohio	1.6 (1.3-1.9)	1.0 (0.8-1.2)	0.3 (0.2-0.5)	0.8 (0.7-0.9)	0.8 (0.7-1.0)	0.7 (0.6-0.9)
Oklahoma	1.3 (0.9-1.9)	1.1 (0.8-1.6)	0.4 (0.1-1.8)	0.8 (0.6-1.2)	1.1 (0.7-1.7)	0.6 (0.3-1.2)
Oregon	1.4 (0.9-2.0)	0.9 (0.5-1.5)	0.2 (0.1-1.0)	0.7 (0.5-1.0)	1.0 (0.6-1.6)	0.4 (0.2-0.7)
Pennsylvania	1.6 (1.3-1.9)	0.9 (0.7-1.1)	0.2 (0.1-0.4)	0.7 (0.6-0.8)	0.8 (0.6-1.0)	0.5 (0.4-0.7)
Rhode Island	1.3 (0.9-2.0)	1.1 (0.8-1.6)	0.1 (0.0-0.8)	0.7 (0.5-1.0)	0.8 (0.5-1.3)	0.7 (0.5-0.9)
South Carolina	1.2 (0.8-1.9)	0.8 (0.5-1.4)	0.4 (0.1-1.3)	0.7 (0.5-1.1)	0.8 (0.4-1.4)	0.6 (0.4-1.1)
South Dakota	0.6 (0.3-1.2)	0.1 (0.0-0.6)	0.1 (0.0-0.8)	0.2 (0.1-0.4)	0.1 (0.0-0.4)	0.2 (0.1-0.6)
Tennessee	2.4 (1.8-3.2)	1.0 (0.6-1.5)	0.3 (0.1-0.8)	0.9 (0.6-1.2)	1.1 (0.8-1.5)	0.7 (0.4-1.1)
Texas	0.8 (0.6-1.0)	0.4 (0.3-0.6)	0.1 (0.1-0.3)	0.4 (0.3-0.5)	0.4 (0.3-0.5)	0.4 (0.3-0.5)
Utah	1.2 (0.8-1.8)	0.8 (0.5-1.3)	0.1 (0.0-0.4)	0.6 (0.5-0.9)	0.6 (0.4-1.1)	0.6 (0.4-1.0)
Vermont	2.1 (1.6-2.7)	0.8 (0.6-1.3)	0.1 (0.0-0.6)	0.7 (0.5-0.9)	0.8 (0.5-1.1)	0.6 (0.4-1.0)
Virginia	1.0 (0.7-1.5)	0.4 (0.2-0.7)	0.1 (0.0-0.8)	0.4 (0.2-0.6)	0.3 (0.2-0.5)	0.4 (0.2-0.8)
Washington	1.1 (0.8-1.6)	0.9 (0.5-1.5)	0.1 (0.0-0.4)	0.6 (0.4-0.9)	0.7 (0.4-1.1)	0.5 (0.3-0.8)
West Virginia	3.3 (2.7-4.1)	1.4 (0.9-2.1)	0.4 (0.1-1.0)	1.2 (0.9-1.5)	1.3 (0.9-1.8)	1.0 (0.7-1.5)
Wisconsin	1.2 (0.8-1.8)	0.5 (0.3-0.8)	0.3 (0.1-1.2)	0.5 (0.3-0.7)	0.4 (0.3-0.7)	0.5 (0.3-1.1)
Wyoming	0.9 (0.6-1.3)	0.4 (0.2-0.8)	0.1 (0.0-0.9)	0.3 (0.2-0.5)	0.4 (0.2-0.7)	0.3 (0.1-0.7)

Supplement S2: Descriptive statistics across states

Table S4. Descriptive statistics across states for opioids variables.

	Mean	SD	Median	Range	Min	Max
Opioid death rates*	10.4	5.7	9.3	28.4	3.2	31.6
Opioid dependence	0.6	0.2	0.7	1.1	0.2	1.3
Heroin dependence	0.1	0.1	0.1	0.4	<0.1	0.4
NMPO dependence	0.6	0.2	0.6	1.0	0.2	1.2

* per 100,000

Table S5. Descriptive statistics across states for socio-demographic factors.

	Mean	SD	Median	Range	Min	Max
GDP/capita [a]	49,168	18,564	46,458	133,140	31,741	164,881
Mean personal income [b]	40,708	6,869	39,384	31,707	30,678	62,384
High school completion [c]	87.9	3.1	88.8	10.9	81.5	92.4
Rurality [d]	0.3	0.2	0.3	0.8	0.0	0.8
Unemployment rate [e]	6.4	1.3	6.5	5.8	3.3	9.1

[a] Average GDP/capita over 2005-2014; [b] Average mean personal income over 2005-2014; [c] Average % of residents who completed high school over 2005-2014; [d] % rural in 2010; (rural defined as city/town with <2,500 pop); [e] Average unemployment rate over 2005-2014; [f] Pooled prevalence of dependence across 2005-2014 of each state; NMPO: non-medical prescription opioids.
